# A New Indole Derivative Decreased *SALL4* Gene Expression in Acute Promyelocytic Leukemia Cell Line (NB4)

**DOI:** 10.22034/ibj.22.2.99

**Published:** 2018-03

**Authors:** Zahra Sheikhrezaei, Parisa Heydari, Alireza Farsinezhad, Ahmad Fatemi, Soudeh Khanamani Falahati-Pour, Shokoofeh Darakhshan, Mojgan Noroozi Karimabad, Ali Darekordi, Hossein Khorramdelazad, Gholamhossein Hassanshahi

**Affiliations:** 1 Department of Hematology and Medical Laboratory Sciences, Faculty of Allied Medical Sciences, Kerman University of Medical Sciences, Kerman, Iran; 2 Pistachio Safety Research Center, Rafsanjan University of Medical Sciences, Rafsanjan, Iran; 3 Department of Pediatrics, Rafsanjan University of Medical Sciences, Rafsanjan, Iran; 4 Molecular Medicine Research Center, Rafsanjan University of Medical Sciences, Rafsanjan, Iran; 5 Department of Chemistry, Faculty of Science, Vali-E-Asr University of Rafsanjan, Rafsanjan, Iran; 6 Department of Immunology, Rafsanjan University of Medical Sciences, Rafsanjan, Iran

**Keywords:** SALL4 protein, Indoles, Leukemia, Acute promyelocytic

## Abstract

**Background:**

Acute myeloblastic leukemia (AML) is a clonal disorder due to bone marrow failure and uncontrolled proliferation of myeloid lineage. Acute promyelocytic leukemia (APL) is a subtype of AML. Heterocyclic compounds, such as indole, are considered as attractive candidates for cancer therapy, due to their abundance in nature and known biological activity. Sal-like protein (SALL4) is a zinc finger transcription factor involving in the multi-potency of stem cells, in the NB4 cell line. This study was aimed to evaluate the effects of basal indole and its new derivative, 2-(1-((2, 4-Aril)imino)-2,2,2-trifluoroethyl) phenyl-1H Indole-3- carbaldehyde (TFPHC), on the expression of *SALL4*.

**Methods:**

Cells were cultured and treated with different concentrations (75, 150, and 300 µg/mL) of the new indole derivative and DMSO, as a vehicle control, for 24 and 48 hours. Cell proliferation was evaluated by using Trypan blue exclusion and MTT assays. The percentage of apoptotic cells was determined by flowcytometry analysis using the Annexin V/PI apoptosis detection kit; mRNA expression of *SALL4* was studied using absolute quantitative RT-PCR.

**Results:**

Our findings demonstrated the effects of new indole derivatives on *SALL4* mRNA expression. Expression of *SALL4* mRNA was significantly decreased at 75, 150, and 300 µg/mL concentrations.

**Conclusion:**

SALL4 plays a role in the survival of APL cells. *SALL4* expression could be suppressed by the novel indole derivative. Additionally, *SALL4* gene suppression can serve as a target in APL therapy.

## INTRODUCTION

Acute leukemia, based on the cellular origin, is categorized into two various divisions, including acute lymphoblastic leukemia (ALL) and acute myeloblastic leukemia (AML). ALL and AML are further divided into different subcategories. Acute promyelocytic leukemia (APL) is a subtype of AML and comprises 4.8%-34% of AML[1]. There is no investigation reporting the exact prevalence of AML in Iran; however, a previous study has shown that the prevalence of the disease is high within the country[2]. Although promising advances have been made specifically via chemotherapy and other supportive cares, remission occurs only in 70-90% of patients with AML[3-5]. Chemotherapy is associated with different side effects varying from nausea and mouth sores to vomiting[4]. Indole-3-carbinol (I3C), found in vegetables, especially in the cruciferous family, has been reported to be beneficial to animal models of cancers[6-8]. There are a few studies addressing the anticancer activity of indole derivatives[9,10]. In the present study, a novel indole derivative was synthesized by our group, and its regulatory effects on the expression of Sal-like protein (SALL4) in NB4 cells were further examined.

*SALL4* is a zinc finger transcription factor encoded by a member of the spalt-like gene family and regarded as a key factor for the maintenance of pluripotency in embryonic stem cells[11,12]. In normal situation, *SALL4* is expressed in bone marrow stem cells but down-regulated in mature blood cells[13]. *SALL4*, as one of the numerable genes, has been indicated to be able to bridge the unique properties of stem cells and malignancies. Unlike its absence or decreased level in most adult tissues, *SALL4* is re-expressed in various human tumors, including hematologic malignancies, as well as liver, gastric, lung, endometrial, and breast cancers[14]. *SALL4* also has an antagonistic function in normal hematopoiesis and leukemia and in proliferation and differentiation of normal hemato-poiesis. However, suppression of the *SALL4* gene in leukemia leads to the induction of apoptosis without notable effects on differentiation[15]. Altogether, these data confirm the important role of *SALL4*, as a cell survival factor, in tumor cells[16-18]. Considering the above issues, we aimed to examine the effects of both basal indole and 2-(1-((2,4-Aril)imino)-2,2,2-trifluoroethyl) phenyl-1H Indole-3-carbaldehyde (TFPHC), as a synthetic novel indole derivative on the expression of *SALL4* in NB4 cells.

## MATERIAL AND METHODS

### Preparation of TFPHC

**Fig. 1:**
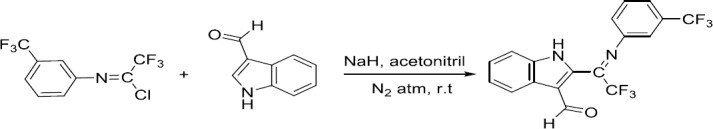
Structure of 2-(1-((2,4-Aril)imino)-2,2,2-trifluoroethyl) phenyl-1H Indole-3-carbaldehyde

The chemical compound was synthesized using the following procedure. A dry, two-necked, 50-mL round-bottomed flask equipped with a nitrogen inlet was charged with 5 mL dry acetonitrile, 0.145 g (1.0 mmol) I3C, and 0.24 g (1.0 mmol) NaH. The resultant solution was stirred under nitrogen atmosphere at room temperature for 30 min. Afterwards, a solution of 2,2,2-trifluoro-N-(3-(trifluoromethyl)phenyl) acetimidoyl chloride (1.0 mmol; Sigma, USA) was added gently and dropwise via a syringe. The mixture was stirred under the N2 atmosphere at room temperature for 20 h and then was filtered. The products obtained from I3C were purified by recrystallization from ethanol (twice; Fig. 1).

The compound was obtained as a white solid, melting point = 114-116 °C, Yield = 82%, Fourier transform infrared spectroscopy (KBr) υ_max_ = 1698, 1673, 1557 cm^-1^. ^1^H-NMR (DMSO-d_6_ 500 MHz) δ = 10.03 (s, 1H), 8.65 (s, 1H), 8.01 (s, 1H), 7.39 (m, 2H), 7.31 (m, 2H), 7.16 (m, 3H) ppm. ^19^F-NMR (CFCl_3_ 475 MHz) δ = -70.852, -62.098 ppm. Anal.Calcd for C_18_H_10_F_6_N_2_O (384.28): C, 56.26; H, 2.62; N, 7.29%. Found: C, 56.34; H, 2.73; N, 7.14%[1].

### Cell culture

The NB4 cell line was purchased from the National Cell Bank of Iran (Pasteur Institute of Iran, Tehran, Iran). Cells were seeded (1 × 10^6^ cells/well) into RPMI-1640 (Gibco Laboratories, Grand Island, NY, USA) containing 10% heat-inactivated fetal bovine serum (Gibco Laboratories, Grand Island, NY, USA) supplemented with 100 IU/mL penicillin and 100 μg/mL streptomycin in a 37ºC humidified incubator with 95% O_2_-5% CO_2_. With appropriate confluence, cells were subjected to passage and then treated with TFPHC that was already dissolved in cell culture medium.

### Cell viability assay

#### MTT assay

This colorimetric assay determines the MTT [3-(4,5-dimethylthiazolyl)-2,5-diphenyl-tetrazolium bromide] reduction. The MTT technique is based on the mitochondrial dehydrogenase activity to generate blue formazan product, reflecting the normal activities of mitochondria, which facilitates the measurement of upcoming cytotoxicity and cell viability.

NB4 cells were seeded at a density of 1 × 10^4^ cells/well into a 96-well plate. The cells were then treated with different concentrations of the novel indole derivative TFPHC (75, 150, and 300 µg/mL), the vehicle control (DMSO), as well as the similar doses of basal indole. After incubation for 24 and 48 hours, the MTT reagent (5 mg/L) was added to each well and incubated for further 4 h. The supernatant was replaced by DMSO, and the relative absorbance was read at 570 nm using a microplate scanning spectrophotometer (ELISA reader, Bio Tek EIK 808, USA). The numbers of viable cells were calculated using appropriate controls. The mean ± SD values are shown from three independent experiments. The inhibition rates were also calculated according to the following formula: Inhibition rate = absorbance value of control group-absorbance value of test group/absorbance value of control group × 100%

#### Trypan blue-based cell viability assay

NB4 cells were seeded onto a 6-well plate at a density of 1 × 10^4^ cells/well. Briefly, the cells were treated with different concentrations of the novel indole derivative TFPHC (75, 150, and 300 µg/mL), the vehicle control (DMSO), as well as the similar doses of basal indole. After incubation for 24 and 48 hours, the number of viable cells in each well was counted under a microscope using a hemocytometer.

### Flowcytometric-based cell analysis of apoptosis

Annexin V-FITC/PI staining was used to determine the apoptotic rates. Following 24 and 48 hours of incubation with various concentrations of the new indole compound, NB4 cells of different groups were collected and transferred into the 5-mL plastic tubes and washed twice with cold PBS. The apoptosis was detected using FITC Annexin V/PI Apoptosis Detection Kit (eBioscience, USA) according to the manufacturer’s instructions using a flow cytometry machine (BD FACSCalibur, USA).

### RNA extraction and cDNA synthesis

The total RNA of cells was extracted using the Trizol™ reagent (Invitrogen, USA) according to the manufacturer’s protocol (Invitrogen, USA). The purity and fidelity of RNA were checked by evaluating optical density (calculation of 260/280 nm ratio) and running onto agarose gel, respectively. The first-strand cDNA was generated using 2 µg total RNA by a high-capacity cDNA Reverse Transcription Kit (Thermo Scientific, USA). The real-time PCR analysis was conducted by application of SYBR Green I Master Mix PCR (GeNet Bio, Korea). Reactions were run using a real-time system (Bio-Rad Company, USA). The software vector NTI was used to design the specific primers for *SALL4*; ß-actin was used as a house keeping gene. The sequences of primers used in this study were 5’ ATTTGTGGCGGAGAGG3’ (*SALL4* forward) and 5’ ACCCCAGCACATCAACTC 3’ (*SALL4* reverse) and 5’GGGCATGGGTCAGAAGG ATT 3’ (β-actin forward), 5’ CGCAGCTCATTGTAG AAGGT 3’ (β-actin reverse).

### Statistical analysis

Student’s *t*-test was applied to analyze the data using SPSS software (version 21). All experiments were performed three times, and all of the acquired results were presented as the mean ± SEM. *P* values less than 0.05 were considered to be significant statistically.

## RESULTS

### Synthesis of a new indole compound

The structure-activity analysis of I3C indicated that the substituents linked to nitrogen atom in the indole ring, which inhibits dehydration and the formation of the reactive indolenine, can increase the potency. It stabilizes the compound and prevents the formation of oligomeric self-condensation products. In contrast, the C-3 hydroxy methyl substituent on the indole ring was derived for the compound biological functions. Further, the increased number of carbons in the N-alkoxy derivatives led to the considerably increased potency, purposing that the hydrophobic properties of the substituents at this indole ring positions were created to evaluate the potency of the generated molecules. In this investigation, we designed and synthesized a new stable indole-3-carbaldehyde derivative containing two trifluoromethyl groups and one phenyl ring that increases hydrophobic and lipophilicity properties. The elevated stability of carbaldehyde, compared to I3C, may depend on the steric effect of bulky addition of the CF3 groups and substituents, which hampers oligomerization of TFPHC action formed under protonation. Hence, the biological effects of TFPHC are independent of its conversation into a derivative. To compare the efficacies of the anti-proliferative activation of TFPHC and the parental compound (I3C), NB4 cells were treated with increasing concentrations of indole for 24 and 48 hours. Our results suggested that the TFPHC has potent anti-proliferative activities.

### Examination of NB4 cell viability by the MTT assay

**Fig. 2:**
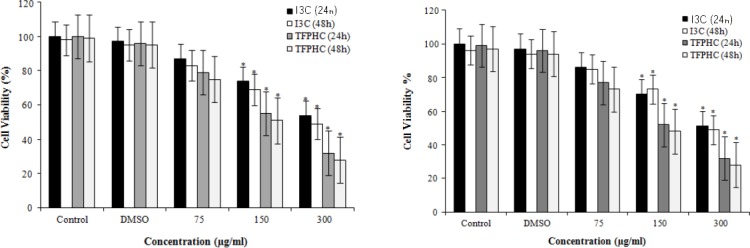
Cytotoxic effects of basal indole and TFPHC plus DMSO on NB4 cell line. (A) Analysis of MTT assay-based viability of cultured NB4 cells. NB4 cells were cultured in the presence of various concentrations of basal indole and TFPHC plus DMSO for 24 and 48 hours. Control cells were remained untreated. Cells were subjected to MTT assay for examination of their viability. (B) Analysis of Trypan-blue based-assay viability of cultured NB4 cells. NB4 cells were cultured in presence of various concentrations of basal indole and TFPHC plus DMSO for 24 and 48 hours. Control cells were remained untreated. At indicated time points, the cells were subjected to staining with Trypan-blue, and the ratio of viable to dead cells was calculated. TFPHC, 2-(1-((2, 4-Aril)imino)-2,2,2-trifluoroethyl) phenyl 1H Indole-3- carbaldehyde; I3C, indole-3-carbaldehyde; * shows significant difference with control DMSO and 75 µg/mL dose.

The MTT assay was used to evaluate the inhibitory effects of new indole derivative on the growth of NB4 cells. NB4 cells were incubated with different concentrations of new indole derivative (0–300 µg/mL) for 24 and 48 hours. The results demonstrated that new indole derivative led to decreased cell viability in NB4 cells in a time and dose-dependent manner (*p* < 0.05; Fig. 2A).

### Examination of NB4 cell viability by Trypan blue exclusion

In Trypan blue analysis, a significant difference in cell viability decline was observed between various concentrations of new indole derivative after 24 and 48 hours (*p* < 0.05; Fig. 2B).

### Examination of NB4 cell viability by flowcytometry

**Fig. 3:**
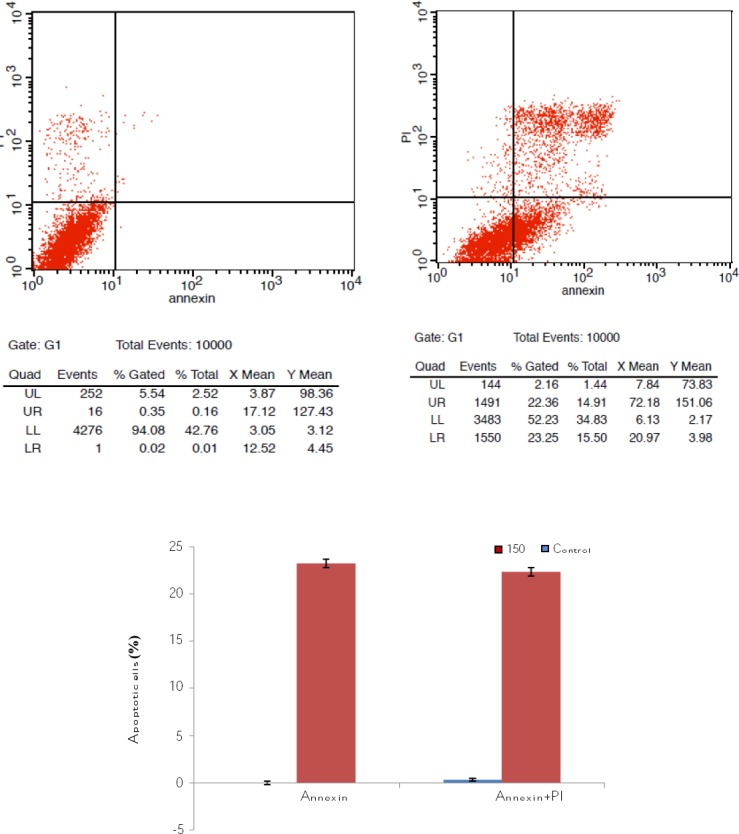
Induction of apoptosis in NB4 cells after treatment with TFPHC in comparison with untreated cells after 24 and 48 at IC50 concentrations. NB4 cells were treated with basal indole and TFPHC for 24 h and 48 h. Cells were stained with Annexin V-fluorescein isothiocyanate (FITC) and propidium iodide (PI). Subsequently, apoptotic and necrotic cells were quantified by flow cytometry. Different subpopulations were defined as UL, Annexin V-negative but PI-positive, i.e. necrotic cells; UR, Annexin V/PI-double positive, i.e. late apoptotic cells; LL, Annexin V/PI-double negative, i.e. normal live cells; and LR, Annexin V-positive but PI-negative, i.e. early apoptotic cells. UL, upper left; UR, upper right; LR, lower right; LL, lower left

To examine the apoptosis, NB4 cells were treated with novel indole derivatives in IC50 concentrations for 24 and 48 hours. At the indicated time points, the treated cells were harvested and stained with annexin V and propidium iodide, and the apoptotic effect of new indole derivative on NB4 cells was detected by flowcytometry. The flowcytometry analysis revealed that the new indole derivative was able to induce apoptosis in NB4 cells, as compared to the control. The normal viable cells were observed in the LL (lower left) region. The early apoptotic cells were determined in LR (lower right) and the late apoptosis and necrosis cells, appeared mainly in the UR (upper right) and UL (upper left) regions, respectively (Fig. 3).

### Evaluation of mRNA expression of SALL4

**Fig. 4:**
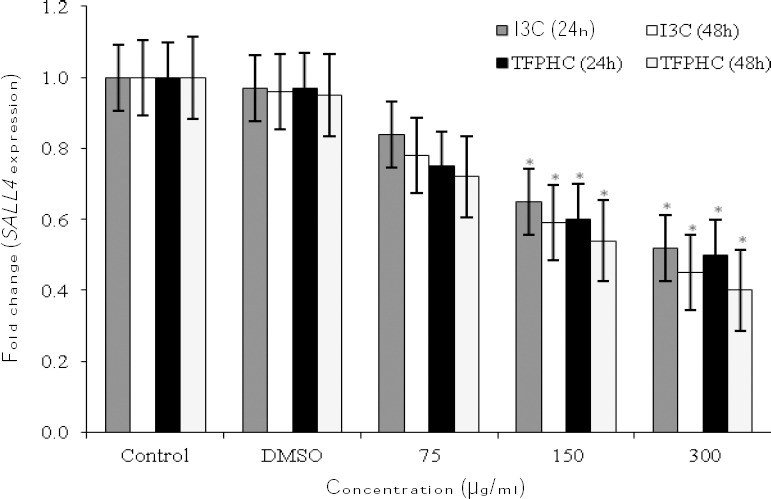
Expression of *SALL4* by NB4 cells in the presence of basal indole and TFPHC following 24 h and 48 h of culture. Following 24 h of culture, cells were harvested and subjected to mRNA expression analysis. The cDNA was synthesized and real-time PCR was performed. The mRNA expression of *SALL4* was then calculated against β-actin (as the housekeeping gene). TFPHC, 2-(1-((2, 4-Aril)imino)-2,2,2-trifluoroethyl) phenyl 1H Indole-3-carbaldehyde; I3C, indole-3-carbaldehyde

Results of the present study showed that the expression of *SALL4* in I3C and TFPHC derivative-treated cells was down-regulated. The mRNA level of *SALL4* was significantly decreased by 1.5fold after 24 hours being exposed to I3C at the dose of 300 µg/ml (*p* < 0.05; Fig. 4). The mRNA expression of *SALL4* was also down-regulated by threefold when the cells were exposed to 15 µg/ml and 300 µg/ml of TFPHC for 24 hours (*p* < 0.05; Fig. 4). We observed that the expression of *SALL4* mRNA was down-regulated after 48 hours in response to I3C and TFPHC (concentrations of 150 and 300 µg/ml) by 2fold and 2.5fold, respectively

## DISCUSSION

*SALL4* is expressed in embryonic cells and plays important roles in the maintenance of pluripotency in human embryonic stem cells[12,19-21]. In human normal bone marrow, *SALL4* is expressed in germ cells while being almost undetectable in other adult tissues. In other words, in normal bone marrow cells, *SALL4* has a key function in hematopoietic differentiation. The present study, for the first time, showed the regulatory effects of both indole and its syntethetic derivative, TFPHC, *in vitro* on the expression of *SALL4*, as a transcription factor involving in the pluri-potency of stem cells. *SALL4* has been shown to be able to aberrantly expressed in different tumor types and hematological malignancies and is involved in leukemogenesis and to have a role in cell death, cancer,

DNA replication/repair, and cell cycle[22]. *SALL4* has also demonstrated to serve as an upstream target for expression of an array of apoptosis genes, including TNF, TP53, PTEN, CARD9, CARD11, CYCS, and LTA, and apoptosis inhibiting genes such as Bmi-1, BCL2, XIAP, DAD1, and TEGT. Cellular apoptosis occurred following the knocking-down of SALL4[23].

AML is amongst the most frequent acute leukemias with an increased incidence with age[24]. The pathogenesis of AML involves mutations in critical genes involved in normal cell development, cellular survival, proliferation, maturation and apoptosis[25]. The complexity of the molecular and cellular structure in AML may explain the reason why advancing the treatment of AML has been an important challenge. Previous studies on different cell lines have shown that the I3C is able to induce potent anti-proliferative activities[26,27]. Our results showed that the expression of *SALL4* was significantly down-regulated in the presence of indole and its derivative as compared with DMSO and control in a dose-dependent fashion; however, the indole derivative was shown to be more potent in several folds when compared to indole itself. Because there was no comparable study within literature concerning the expression of *SALL4*, these data provide novel evidence that *SALL4* serves as an upstream target for pro-/anti-apoptosis genes. Therefore, these findings are in agreement with the results of investigators who reported the up-regulated levels of pro-apoptotic genes, including TNF, TP53, PTEN, CARD9, CARD11, CYCS, LTA, as well as Bmi-1, BCL2, XIAP, DAD1, and TEGT[23]. Several studies approached the enhancing potencies of cytotoxicity as well as induction of apoptosis in cancer cells by synthesizing more potent I3C dimerized products[28-30]. This novel I3C derivative is one of the most potent synthetic derivatives of I3C that results in several folds increased tendency of the cell cycle arrest[10] and apoptosis[31] in NB4 cancer cells. Our recent data also demonstrated that this novel compound (TFPHC) has down-regulated both OCT4 and NANOG, the genes which are involved in cellular immortality[32].

As mentioned above, SALL4 is a key regulator of cell growth and apoptosis in leukemic cells[33]; hence, it is able to be targeted as a promising therapeutic target. In the present study, NB4 cells were treated with 75, 150, and 300 µg/mL of TFPHC for 24 hours. Further, RNA was extracted from the cultured cells, and cDNA was synthesized, followed by RNA analysis using real-time PCR. According to our data, the down-regulation of *SALL4* in the presence of TFPHC (the new indole derivative) may explain a mechanism by which the compound prevents the proliferation of NB4 cells (via decreasing the *SALL4* expression).

The role of *SALL4* in APL is unknown, but this study showed that this gene may probably be involved in pathophysiology of this disease, and TFPHC is able to reduce the expression of *SALL4*. Further studies are required to address the exact role of *SALL4* in APL. In addition, this study demonstrated that targeting of *SALL4* by TFPHC can possibly be used as a promising treatment for APL.
